# Type and Distribution of Sensilla in the Antennae of the Red Clover Root Borer, *Hylastinus obscurus*

**DOI:** 10.1673/031.013.13301

**Published:** 2013-11-23

**Authors:** Rubén Palma, Ana Mutis, Rufus Isaacs, Andrés Quiroz

**Affiliations:** 1 Doctorate Program in Sciences and Natural Resources. Universidad de La Frontera, Temuco 4811230, Araucanía, Chile; 2 Departamento de Ciencias Químicas y Recursos Naturales, Universidad de La Frontera, Temuco 4811230, Araucanía, Chile; 3 Department of Entomology, Michigan State University, East Lansing, MI 48824; 4 Current address: Laboratorio de Interacciones Insecto-Planta, Universidad de Talca, Talca, Chile

**Keywords:** chemoreception, scanning electron microscopy, Scolytinae, transmission electron microscopy

## Abstract

In order to determine the type, distribution, and structures of sensilla, the antennae of the red clover root borer, *Hylastinus obscurus* Marsham (Coleoptera: Curculionidae: Scolytinae), were examined by light and electron microscopy (both scanning and transmission). Four different types of sensilla were identified in the club, and one type of chaetica was found in the scape and funicle of both male and female individuals. Chaetica and basiconica were the most abundant sensilla types in the club. They were present in the three sensory bands described, totaling approximately 80% of sensilla in the antennal club of *H. obscurus*. Chaetica were predominantly mechanoreceptors, although gustatory function could not be excluded. Basiconica forms showed characteristics typical of olfactory sensilla. Trichoidea were not found in the proximal sensory band, and they exhibited abundant pores, suggesting olfactory function. Styloconica were the least abundant sensillum type, and their shape was similar to that reported as having hygro- and thermoreceptor functions. There was no difference in the relative abundance of antennal sensilla between males and females. Finally, the sensillar configuration and abundance of receptors in the *H. obscurus* antennae suggest that these sensilla have chemoreceptive and other functions.

## Introduction

Scolytinae is an important insect group that comprises approximately 6,000 species (Grégoire and Evans 2004). These beetles attack healthy trees that they girdle and kill and feed on fungi that they cultivate in tunnels (Gillott 2003). They occupy a wide range of niches among various herbaceous and woody plants (Zúñiga et al. 2002). The red clover root borer, *Hylastinus obscurus* Marsham (Coleoptera: Curculionidae), is a scolytine pest of red clover, *Trifolium pratense*, (Fabales: Fabaceae), a widespread leguminous forage crop. This beetle feeds on roots and is considered to be the primary cause of the decline of this plant (Quiroz et al. 2005). As with other Scolytinae species, the importance of chemical stimuli has been studied to understand this beetle's behavior (see Byers 1989). Tapia et al. (2007) reported the preference of *H. obscurus* for volatiles extracted from 1.5-year-old red clover roots compared to those extracted from roots of 2.5-year-old plants, suggesting that *E*- 2-hexenal attracted the insect, whereas limonene repelled it. Manosalva et al. (2011) noted the attractive effect of several long-chain fatty acids found in roots of 9-month-old clover plants on these beetles.

It is well known that the olfactory system is the primary sense that insects use in analyzing the environment (Krieger et al. 1997; Ryan 2002) in crucial tasks such as finding food, nesting, mating, and identifying conspecifics (Picimbon 2003; Larsson and Svensson 2005; Hallem et al. 2006). The antennae contain a high concentration of olfactory chemoreceptors (Klowden 2007), which confer the ability to discriminate between physiologically irrelevant compounds and essential chemical signals (Leal 2003). Moeck (1968) described the antennal sensilla of *Trypodendron lineatum*, and Payne et al. (1973) noted clear differences between the antennae of 16 Scolytinae species. The utility of this information exceeds the purely morphological concerns because it can provide a foundation for electrophysiological studies (Whitehead 1981) and also verify the use of the chemosensory system as an odor detector for identifying relevant compounds (Larsson and Svensson 2004).

Although knowledge of the chemical signals involved in the behavior of *H. obscurus* has increased in the recent years, questions remain about this species' antennal morphology. Understanding the detailed structure of antennal morphology would be highly valuable, not only to gain a better overview of this insect's biology but also for performing electrophysiological studies to elucidate the chemical ecology of this important crop pest.

## Materials and Methods

### Insects

Adult *H. obscurus* individuals were isolated from 2-year-old red clover roots collected from red clover plots in the Regional Research Center INIA-Carillanca, Araucanía, Chile, where the species is highly abundant (Klein and Waterhouse 2000). The insects were separated and stored individually in Petri dishes. Then they were frozen at -20° C for 10 hr before sample preparation. The whole heads and antennae were cut using a scalpel, and the sex of individuals was determined under a light microscope, following the methodology reported by Carrillo et al. (1978).

### Microscopy

Scanning electron microscopy was carried out following the methodology employed in the Electronic Microscopy Laboratory of Universidad de Concepción, Chile. Heads and antennae of 20 previously frozen beetles were immersed in a 15% v/v ethanol solution and sonicated for 30 sec. The samples were then dehydrated by placement in 30, 50, 70, 90, and 99% v/v ethanol solutions for 3 min each, and they were then left to dry overnight at room temperature. Finally, the samples were put in a sample holder, treated in an Edwards S150 Sputter Coater critical point dryer (http://www.ipk-gatersleben.de) for 20 min, and gold-coated. Three to five males and females were observed using a LEO 420 microscope (Carl Zeiss SMT, http://www.smt.zeiss.com).

The samples for transmission electron microscopy were obtained in the same way as those used in scanning electron microscopy. The tissues were immersed in a 3% v/v glutaraldehyde solution for 24 hr and transferred to 0.1% w/v cacodylate buffer for 4 hr at room temperature. Then they were fixed in a 1% w/v osmium tetroxide solution and sequentially dehydrated in acetone to be embedded in a resin composed of 1:3 polypropylene oxide and araldite. Finally, they were mounted in gel capsules and oven-dried for 48 hr before being cut in 40–50 nm thickness slides using a DuPont MT500 ultramicrotome (DuPont, http://www.dupont.com). The samples were observed using a JEM-1200 EX II microscope (JEOL, http://www.jeoleuro.com) at 80 kV.

### Image and data analysis

The images obtained by scanning and transmission electron microscopy were processed using ImageJ software v. 1.44p (National Institute of Health, http://rsbweb.nih.gov/ij). At least three replicates were used to calculate the means of size and sensilla number. To classify the sensilla, the terminology of Borg and Norris (1971) and Ryan (2002) was applied. Then, the data were analyzed using Student *t*-tests using StatsDirect v.2.7.8 (StatsDirect Ltd., http://www.statsdirect.com).

## Results

### The antennae

**Figure 1.**
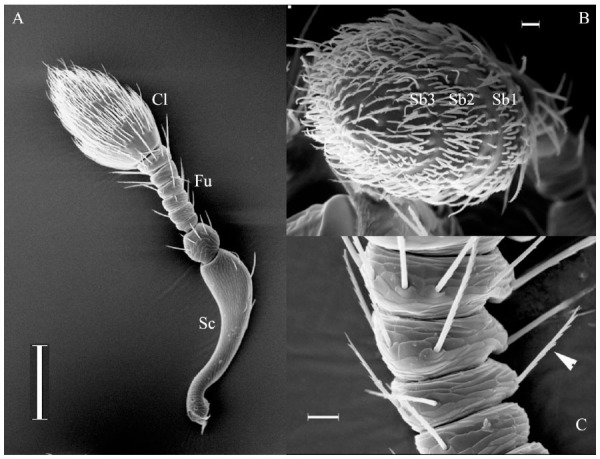
Antenna of *Hylastinus obscurus*. (A) General overview showing the scape (Sc), the funicle (Fu) formed by six segments, and the club (Cl). (B) The spatial distribution of sensilla, which form three sensory bands (Sb1, Sb2, and Sb3). (C) Close-up of the antenites forming the funicle, showing some type I chaetica sensilla (arrows) emerging from the cuticle. Scale bar: A = 100 µm; B = 20 µm; C = 10 µm. High quality figures are available online.

The *H. obscurus* antennae consisted from base to tip of a long and curved scape, followed by a pedicel which was longer and wider than the successive antenites that formed the funicle. The funicle was formed by six non-soldered flagellomeres, which flanked the club, a rigid and round structure composed of fused segments (Figure 1A). The average length of female antennae was 568 ± 65 µm (n = 3), which is slightly longer than the average of 545 ± 31 µm (n=3) the male antennae. The scarce number of sensilla on the scape, pedicel, and funicle contrasted with the abundantly-covered club (Table 2), where the sensilla were distributed in three transverse sensory bands.

Each one of these bands (Figure 1B) was formed by a wide belt of chaetica sensilla in the proximal region, such that the upper part of those sensilla covered the distal narrow belt next to them that was formed primarily by basiconica sensilla.

**Table 1.**
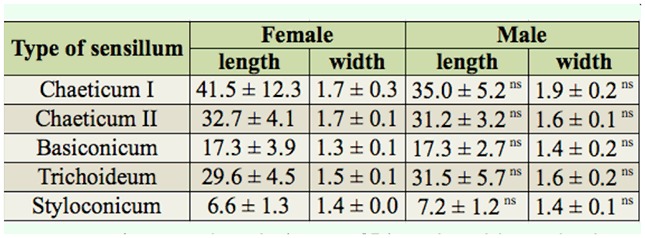
Average length (µm ± SD) and width at the base (µm ± SD) of the different types of sensilla found on the antennae of *Hylastinus obscurus* males and females **. ns No significant differences were found between the sexes according to a Student's *t*-test (*p* > 0.05).

**Table 2.**
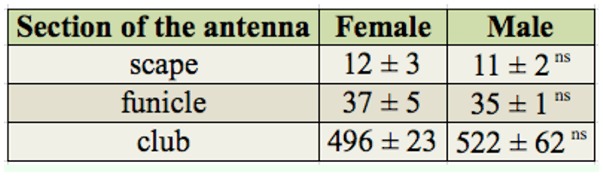
Average number of sensilla (± SD) in the different sections of the *Hylastinus obscurus* antenna. ns No significant differences were found between sexes according to a Student's *t*-test (*p* > 0.05).

**Table 3.**
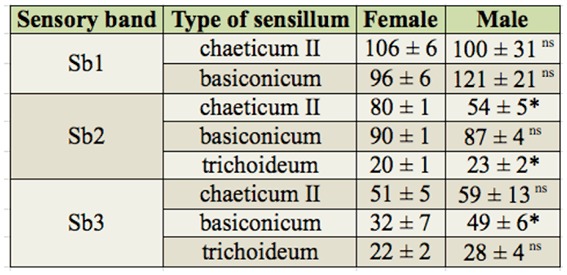
Average number of the three most abundant types of sensilla (± SD) in the antennal club of *Hylastinus obscurus* by sex, sensory band, and type of sensillum. * Significant difference between sexes according to a Student's *t*-test (*p* ≤ 0.05) ns Not significant.

The type and size of sensilla covering the different parts of the antenna were similar in male and female beetles, and no apparent sexual dimorphism was found in these sensitive structures (Table 1).

### Types of sensilla

The numbers of sensilla were significantly higher in the antennal club than the rest of the antenna, and there were no significant differences between males and females (Table 2). A decreasing number of the two most abundant types of sensilla in the antennal club were found from band 1 (Sb1) to band 3 (Sb3) (Table 3). Chaetica were the most abundant sensilla found in the antennal club, and there were significant differences between males and females in the number of sensilla of the three types located in Sb2 and Sb3 (Table 3). In the scape and funicle, the sensilla were clearly distinguishable by their length, which ranged from 28–61 µm, and by their orientation at approximately 45° with respect to the surface (Figure 1C). These sensilla were equipped with a socket (Figure 1C), and they had basal diameters of 1.3–2.3 µm, with a diameter of 0.2 µm at the apex. They were bilaterally branched, the number of spine-like branches varied from 7–20, and they were alternately distributed on the smooth surface of the sensillum. Pores were not observed in the surface.

**Figure 2.**
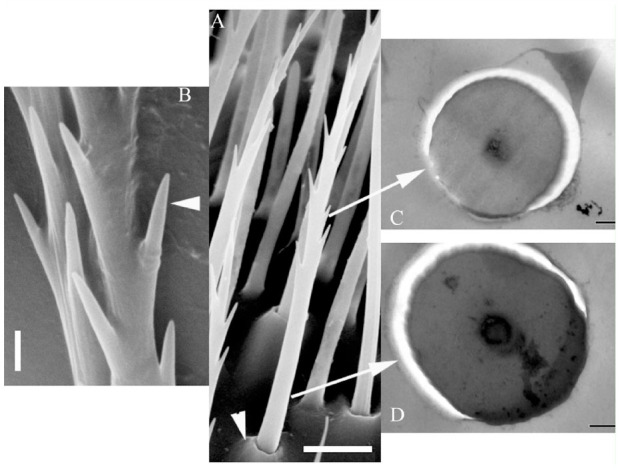
(A) Overview of a type II sensillum chaeticum supported in a socket (arrowhead). (B) Detail of the laterally alternating branches (arrowhead). (C) Transmission electron microscopy image of a transversal cut in the upper part and (D) lower part of the sensillum. Scale bars: A = 5 µm; B = 1 µm; C–D = 0.2 µm. High quality figures are available online.

In the club, the shape of chaetica sensilla was similar to those found in the scape and funicle. They were supported in a socket (Figure 2A) and ranged from 26–37 µm in length. The basal diameter of these sensilla was 1.5–1.7 µm, with a diameter at the apex of 0.3 µm. There were from 5–9 spine-like branches per sensillum. Transmission microscopy showed circular, solid cross sections with no dendritic segments or pores in the wall (Figure 2C, D).

**Figure 3.**
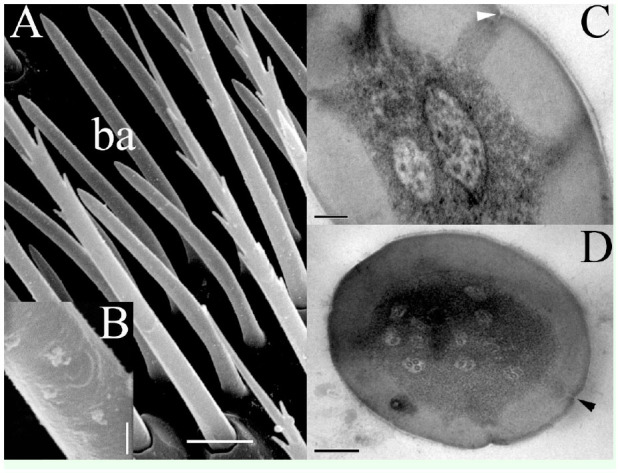
(A) Sensillum basiconicum (ba) in a sensory band. (B) shows the slightly grooved surface of this type of sensilla. (C) In the cross-section, the thick wall is interrupted by spaced pores (arrowhead), which communicate outwardly using three dendrites in the lumen. (D) Several dendritic segments and pores (arrowhead) formed in the upper part of the basiconicum. Scale bars: A = 5 µm; B = 0.5 µm; C = 0.2 µm; D = 0.1 µm. High quality figures are available online.

Basiconica sensilla (Figure 3) were found in the club only and formed a stretched belt in the sensory band in a distal position with respect to the chaetica sensilla. They did not have a socket, but they did have an oblique insertion, which varied in form from straight in the proximal sensory band to bent in the distal sensory band. The external surface was slightly rough (Figure 3B), and the length varied from 9–21 µm, with a basal diameter ranging from 1.3–1.7 µm, finishing with a sharp apex of 0.1 µm. Internally, these sensilla had a thick wall of 0.2 µm, which was disrupted by pores in the upper section that linked the surface to the sensillar lumen (Figure 3C). The center of the sensillar lumen was occupied by dendritic segments with numbers ranging from 2–16.

**Figure 4.**
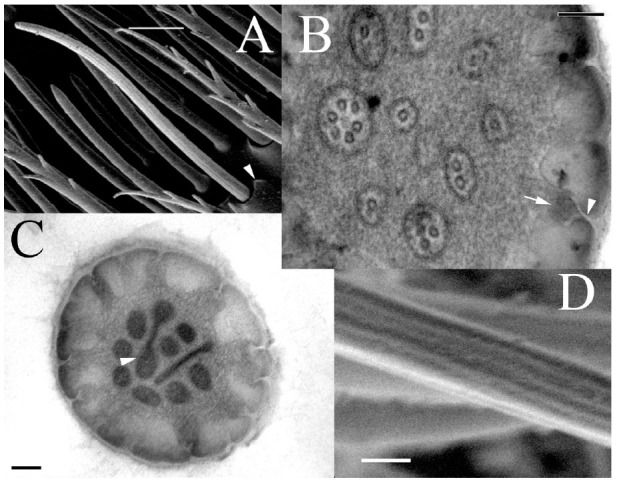
Sensillum trichoideum (A) supported in socket (arrowhead) with a grooved surface (D). (B) Pores formed by short and stretched channels (arrowhead) connecting the surface to the sensillar lumen (arrow), which is filled with dendritic segments. (C) Incompletely formed dendritic segments (arrowhead) appeared in the upper part, and a high density of pores characterized this type of sensillum. Scale bars: A = 5 µm; B = 0.1 µm; C = 0.1 µm; D = 1 µm. High quality figures are available online.

Sensilla trichoidea appeared dispersed in the sensory bands among the chaetica and basiconica sensilla, without a clear pattern of distribution. Their length varied from 20–38 µm. The basal diameter was 1.3–1.9 µm, clearly tapering to a rounded tip of 0.3 µm width. The density of this type of sensillum varied in the different sensory bands, being more abundant in the distal than proximal one (Table 3). Besides the presence of a socket at the base, the most obvious external morphological feature was the rough surface that formed longitudinal strips (Figure 4). Transmission electron microscopy pictures showed a thick wall of 0.2 µm thickness, interrupted by numerous pores, while the center of the sensillar lumen was filled with dendritic segments, which ranged in number from 2–18 (Figure 4).

**Figure 5.**
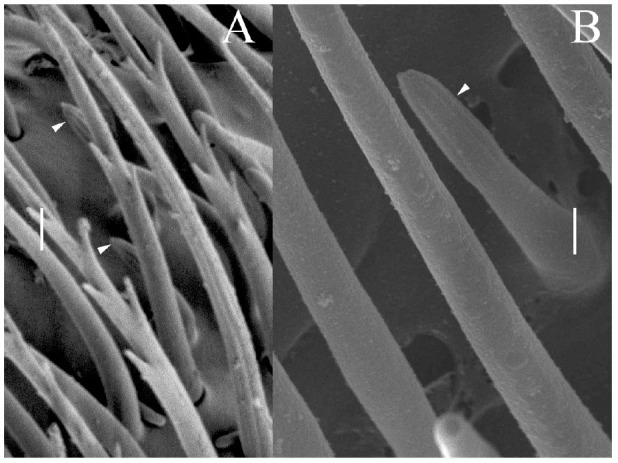
(A) Sensillum styloconicum found in the second sensory band in a male antenna indicated by arrowheads. (B) The finger-shaped pegs in the top of this type of sensillum resemble a bud (arrowhead). Scale bars: A = 2 µm; B = 1 µm. High quality figures are available online.

The styloconic sensilla (Figure 5) were scarce and found only in the distal sensory band (Sb3). Their length varied from 6–8 µm and the basal diameter from 1.3–1.5 µm. There was no socket, and they had a smooth surface, with the upper half formed by approximately 10 finger-shaped pegs, like a bud (Figure 5B).

## Discussion

This study revealed the ultrastructure of the antennae of *H. obscurus*, finding a complex arrangement of multiple sensillum types. This information can be used to guide future electrophysiological investigations of the ability of *H. obscurus* to detect semiochemicals. The antennae of adult insects typically contain various types of sensilla that play important roles in a number of behaviors (Hu et al. 2009). According to reviews of different reports (Borg and Norris 1971; Payne et al. 1973; Dickens and Payne 1978; Andersson et al. 2009; Chen et al. 2010; Wang et al. 2012), the diversity of structure types reported here is relatively common for scolytines, but there is no unified system for naming them, making it difficult to compare results even among antennal systems of closely related species. As noted by Merivee et al. (1999), this type of confusion in sensilla terminology is due to a lack of fixed criteria for discrimination.

Even though the number of antennal sensilla and the diversity of sensilla types vary greatly among different insect species, the relative number and diversity of sensilla found in *H. obscurus* are close in magnitude to those reported previously for other members of Scolytinae, such as *Scolytus multistriatus* (Borg and Norris 1971), *Dendroctonus frontalis* (Dickens and Payne 1978), and *D. ponderosae* (Whitehead 1981). In these species, antennae were also found to contain 148–186 chaetica, 45–308 basiconica, and 140–460 trichoidea sensilla.

For those species for which micrographs are available, the sexes showed no apparent differences in the type, relative number, location, or size of sensilla, as noted in *H. obscurus* (Table 2). There were considerable variations in the range of lengths of the sensilla (Table 1). However, in general, the variations in sensillum length did not appear to correlate with sex, species, or genus, and the sensillum length appeared not to correlate with the size of the antennae (Payne et al. 1973). On the other hand, some differences appeared when the different types of sensilla were compared in each sensory band. These differences have not been noted before in this group of insects, and the meaning of these distinctions remains to be elucidated, especially considering that morphology does not correlate with the function of each type of sensilla (Ryan 2002).

Characterized by their thick wall and long and heavy shape (Ryan 2002), chaetica in the club represented more than 40% of the total sensilla (Table 3). In comparison to those in the scape and funicle, the presence of a socket at the base differentiated the type II chaetica from the type I chaetica. According to Borg and Norris (1971), who studied the antennal club of *S. multistriatus*, chaetica function as mechanoreceptors, enabling the insect to determine the position of the antenna with respect to its surroundings (Payne et al. 1973). The two rows of pegs that projected outward and formed an angle of approximately 120° (Figure 2B) may be considered modifications that allow the detection and transmission of diverse mechanical stimuli (Keil 1997).

Internally, chaetica have a thick wall, and no pores were observed on the surface (Figure 2A). Borg and Norris (1971) reported that the dendrites do not penetrate the seta but remain at the base because of their function. The images of different sections of this type of sensillum in *H. obscurus* (Figure 2C, D) showed that the lumen was not filled with dendritic sections. The lack of both attributes (cuticular pores and dendritic section) led Chen et al. (2010), who studied antennal sensilla of *Dendroctonus valens*, and Wang et al. (2012), who analyzed the antennal ultrastructure of three *Tomicus* species, to hypothesize mechanoreception as the most probable function of chaetica sensilla.

The relative abundance of basiconica sensilla was comparable to that of chaetica, but the basiconica sensilla were compact and formed a sort of palisade, in the same manner as those described previously by Payne et al. (1973) for the antennae of *Pseudohylesinus* sp. and several *Dendroctonus* species. Internally, the sensilla were notable by their thick wall, and they showed pores only on the upper part (Figure 3C, D), which were arranged radially in the cuticular wall, suggesting olfaction as the likely function of this type of sensillum (Klowden 2007). This description agrees with those for basiconica sensilla found in *Tomicus* sp. (Wang et al. 2012) and *Dendroctonus valens* (Chen et al. 2010).

Trichoideum was the third most abundant type of sensillum. Described as long, slender, and hair-like (Ryan 2002), this form dominated in the distal sensory band (Figure 1B) as opposed to the proximal sensory band, where trichidea were not found (Table 3). Internally, they were abundant in pores, forming radial channels connecting the surface to the lumen (Figure 4B, C). The olfactory function is proposed as the most probable. The structure of this sensillum type on *H. obscurus* resembled the sensilla trichodea 3 described by Chen et al. (2010) in *D. valens* and trichodea found in the antenna of *Limonius aeroginosus* (Merivee et al. 1998).

Styloconica sensilla (Figure 5) were quite scarce and difficult to find within the sensory bands of male and female antennae. It was not possible to get any transmission electron microscopy image, which could help to further determine their function. However, their shape and size resemble those reported by Dickens and Payne (1978) as fluted sensilla in *D. frontalis*, also called basiconica sensilla type II in *Semiadalia undecimnotata* (Jourdan et al. 1995) and grooved pegs in *Trogossita japonica* (Usha Rani and Nakamuta 2001), *Callosobrochus chinensis*, and *C. maculatus* (Hu et al. 2009). Bartlet et al. (1999) and Steinbrecht (1989) reported these as thermoand hygrosensitive receptors, but Hu et al. (2009) added chemoreception as another possible function. The reduced number of this type of sensilla in insects compared to the more abundant chemo- and mechanoreceptors was noted by Altner and Loftus (1985).

In conclusion, the antennae of *H. obscurus* did not differ from other species in the bark beetle group. Additionally, males and females of *H. obscurus* did not show clear differences in the type, length, or overall number of antennal structures. Furthermore, the presence of porous sensilla reflected the ability of antennae to perceive chemical stimuli, and these may be the subject of further studies using isolated sensilla to elucidate their particular functions and contributions to the chemical ecology knowledge of this species. The relatively highly abundant mechanoreceptors, especially in the club, should be considered as a potential target of interference by the addition of noise for electroantennographical studies of this borer.
